# Methodological approaches to population based research of screening procedures in the presence of selection bias and exposure measurement error: colonoscopy and colorectal cancer outcomes in Ontario

**DOI:** 10.1186/1471-2288-13-59

**Published:** 2013-04-24

**Authors:** Binu J Jacob, Rinku Sutradhar, Rahim Moineddin, Nancy N Baxter, David R Urbach

**Affiliations:** 1 Division of Support, Systems and Outcomes, Toronto General Hospital, 200 Elizabeth Street, Toronto, ON, M5G 2C4, Canada; 2 Institute of Medical Science, University of Toronto, Toronto, Canada; 3 Institute for Clinical Evaluative Sciences, Toronto, Canada; 4 Dalla Lana School of Public Health, University of Toronto, Toronto, Canada; 5 Department of Family and Community Medicine, University of Toronto, Toronto, Canada; 6 Department of Surgery and Keenan Research Centre, Li Ka Shing Knowledge Institute, St. Michael’s Hospital, Toronto, Canada; 7 Department of Health Policy Management Evaluation, University of Toronto, Toronto, Canada; 8 Cancer Care Ontario, Toronto, ON, Canada; 9 Department of Surgery, University of Toronto, Toronto, Canada

**Keywords:** Colonoscopy, Propensity score analysis, Instrumental variable analysis

## Abstract

**Background:**

The study describes the methodological challenges encountered in an observational study estimating the effectiveness of colonoscopy in reducing colorectal cancer (CRC) incidence and mortality.

**Methods:**

Using Ontario provincial administrative data, we conducted a population-based retrospective cohort study to assess CRC incidence and mortality in a group of average-risk subjects aged 50–74 years who underwent colonoscopy between 1996–2000. We created two study cohorts; unselected and restricted. The unselected cohort consists of subjects aged 50–74 years who were eligible for CRC screening and who had the same primary care physician (PCP) during the period 1996–2000 with at least two years of follow-up. PCPs are general practioners/family physicians who are the main source of health care for Ontarians. The restricted cohort was a nested sample of unselected cohort who were alive and free of CRC as on January 1, 2001 and whose PCPs had at least 10 screen-eligible patients with a colonoscopy referral rate of more than 3%. We compared the outcomes in the two study cohorts; unselected vs. restricted. We then estimated the absolute risk reduction associated with colonoscopy in preventing CRC incidence and mortality in the restricted cohort, using traditional regression analysis, propensity score analysis and instrumental variable analysis.

**Results:**

The unselected cohort (N = 1,341,612) showed that colonoscopy was associated with an increase in CRC incidence (1.61% vs. 4.61%) and mortality (0.36% vs. 1.16%), whereas the restricted cohort (N = 1,089,998) showed that colonoscopy was associated with a reduction in CRC incidence (1.36% vs. 0.84%) and mortality (0.23% vs. 0.15%). For CRC incidence, the absolute risk reduction (ARR) associated with colonoscopy use was 0.52% in an unadjusted model, 0.53% in a multivariate logistic regression model, 0.54% in a propensity score-weighted outcome model, 0.56% in propensity score-matched model, and 0.60% using instrumental variable analysis. For CRC mortality, the ARR was 0.08% in the unadjusted model, multivariate logistic regression model and for a propensity score- weighted outcome model, 0.10% using propensity score matched model and 0.17% using the IVA model.

**Conclusions:**

Colonoscopy use reduced the risk of CRC incidence and mortality in the restricted cohort. The study highlights the importance of appropriate selection of study subjects and use of analytic methods for the evaluation of screening methods using observational data.

## Background

Colorectal cancer (CRC) is the third most common cancer and has the second highest cancer mortality rate among Canadian men and women [1,2]. Colonoscopy is considered an effective tool for CRC screening [3,4] and studies have shown that colonoscopy is associated with a reduction of CRC incidence and mortality, but primarily limited to death due to left sided colon and rectal cancers [5-8]. Due to patient demand [9], reimbursement policies and economic arguments [10,11] and advocacy by specialty organizations [12,13], the use of colonoscopy has increased in the last decade in Canada [14,15]. Despite the increased use of colonoscopy, its benefit at the population-level is still uncertain. Evaluating a screening tool and answering such a policy question using observational data involves many challenges. Colonoscopy is used in Ontario for a variety of purposes including screening, diagnosis and surveillance of CRC. It is difficult to distinguish screening from therapeutic applications of colonoscopy using administrative data. Moreover, persons studied using observational data sources such as electronic administrative data usually differ in baseline characteristics—both measured and unmeasured—which are associated with both treatment assignment as well as their health outcomes. Hence, using population-based health services information to estimate the effectiveness of colonoscopy on the risk of CRC incidence and mortality is prone to various biases. Failure to account for these biases can lead to incorrect estimates of the effectiveness of colonoscopy.

Standard analytic methods such as multivariate logistic regression can be used to adjust observed differences between treatment groups for dichotomous outcomes [16]. Propensity score methods are increasingly used for adjusting selection bias as an alternative to regression adjustment and also in health policy studies [17,18]. Both methods can reduce bias in causal estimates due to observed differences but are still subject to biases from unobserved differences between treatment groups. Instrumental variable methods have been used in health policy studies in the presence of hidden bias [19,20]. While different analytic methods are available to compare the treatment groups in observational studies, it is not clear how different methods yield different findings.

The objective of this paper is to illustrate and compare different analytic methods and study designs in a study to estimate the effect of colonoscopy in reducing incidence and mortality due to CRC using administrative data and cancer registry information from Ontario, Canada.

## Methods

We conducted a population-based retrospective cohort study to evaluate CRC incidence and mortality associated with exposure to colonoscopy in a population of Ontario, Canada who are at risk of CRC. This paper discusses different approaches to patient selection and analytic methods, to illustrate how different methodologies account for the biases that complicate efforts to evaluate a screening test using administrative data in health services research.

### Data sources

We used information from several administrative databases and cancer registry. The Registered Persons Data Base (RPDB) contains the demographic information on all residents eligible for health care in Ontario. The Ontario Health Insurance Plan (OHIP) database contains information on claims for physicians’ services provided to Ontario residents, and includes virtually all medical services provided in Ontario. The Canadian Institute for Health Information–Discharge Abstract Database (CIHI-DAD) contains information on all discharges from acute care facilities for residents of Ontario, including patient demographics, diagnoses, procedures, and discharge status. The Ontario Cancer Registry (OCR) has records of all Ontario residents newly diagnosed with cancer or who died from cancer, and is estimated to be 95% complete [21]. The 2001 Canadian Census files contain aggregated data on socio-economic information of the Canadian population at the census tract level. These databases were linked using an anonymous encrypted number and have very little missing information [22].

The study was approved by the Research Ethics Board of Sunnybrook Health Sciences Centre, Toronto, Canada.

### Study subjects

We performed the study using two cohorts of subjects. First, we created an *unselected cohort* consisting of all residents of Ontario aged 50–74 years during the period 1996–2000 and who appeared to be eligible for CRC screening (no prior diagnosis of CRC, inflammatory bowel disease (IBD), adenomas, or colonic polyps, no previous colon or rectal surgery, and no colonoscopy in the previous 4 years). We included only subjects who had the same primary care physician (PCP) over the 5 years period with at least 2 years of follow-up. Subjects who could not be assigned to an individual PCP using the continuity of care (COC) method [23,24] (14% of the cohort) were excluded from this cohort, During the study period, most referrals to specialists were originated from PCPs. We wanted to include only patients who could be assigned to a PCP as our aim was to capture the variation in PCP’s use of colonoscopy as an instrument. Subjects residing in areas where specialist physicians did not submit fee-for-service claims for colonoscopy (<5% of the cohort) were also excluded. The details of the areas where physicians do not bill directly for services were reported elsewhere [25].

**Figure 1:**
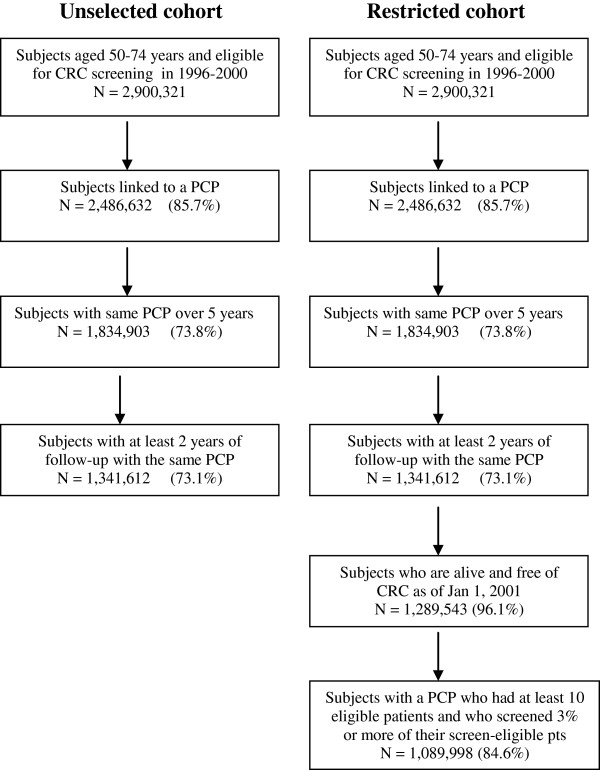
Selection of two cohorts – Unselected cohort vs. Restricted cohort.

There is a problem measuring screening exposure and outcomes of patients during the same time period. It is likely that procedure that have done for diagnostic purposes and on patients with symptoms would result in a diagnosis of CRC. In this case, an observational study would counter-intuitively identify an association between increased colonoscopy use and a higher risk of CRC. Therefore we tried to restrict our cohort and explored the use of a cohort of subjects whose colonoscopy exposure was measured independent of their risk of developing CRC. In the second cohort—the *restricted cohort*—we identified a nested group of subjects from the unselected cohort who were alive and free of CRC on January 1, 2001. We excluded patients of PCPs with fewer than 10 screen-eligible patients and whose PCPs referred colonoscopy in less than 3% of the patients linked to them. This exclusion had done to avoid subjects whose PCPs were not primarily responsible for medical referrals for their patients. Our intention was not to classify patients based on their symptoms, or symptomatic versus asymptomatic subjects. We were interested in studying colonoscopy use among patients in whom CRC screening might be considered. The selection criteria used in the two cohorts is depicted in Figure 1.

### Identification of exposure

The occurrence of any colonoscopy procedure during the period 1996–2000 was the principal exposure. We identified all colonoscopies performed on the subjects using OHIP fee codes. Subjects were classified as the exposed group if there was a receipt of any colonoscopy service at any time during the 5 year period 1996–2000.

### Identification of outcomes

The primary outcomes were cumulative 7-year incidence and 5-year mortality due to CRC. Cohort subjects were followed up to December 31, 2007 for CRC incidence and up to December 31, 2005 for CRC death, to account for delays in ascertaining cause of death. There was virtually no loss to follow-up for the outcomes; subjects were censored at the end of the appropriate follow-up period if they were still alive. Incident CRC and death due to CRC was identified in the OCR.

### Characteristics of study subjects

Subject characteristics included age, sex, household income quintile, comorbidity and rural residence, as measured on 2000. Comorbidity was measured using a modified Charlson comorbidity Index [26,27] from inpatient claims diagnosis codes, based on hospital discharges up to four years prior to their entry into study. Subjects were classified as having no inpatient claims, or being hospitalized with no comorbidities and being hospitalized with one or more comorbidities. Census 2001 data were used to obtain neighborhood household income quintile. Neighborhood income is calculated by Statistics Canada and is available by dissemination area (DA), which is the smallest geographic area for which census data are made available. Each patient’s DA of residence was identified based on their postal code and then linked to the Statistics Canada postal code conversion file to get the neighborhood income [25]. This variable was used as an ecological measure of socioeconomic status of subjects.

### Statistical analysis

We first performed a descriptive analysis of the two study cohorts. We estimated the absolute risk reduction (ARR) and 95% confidence intervals for development of CRC outcomes between those subjects who received a colonoscopy and those who did not.

Since CRC outcomes are influenced not only by use of colonoscopy, but also by many other measured determinants of colonoscopy, we then used logistic regression analysis with generalized estimating equation (GEE) models to adjust for measured confounding factors and the clustering of subjects within PCPs. In addition to colonoscopy exposure, models included the subject level covariates age, sex, Charlson comorbidity score, rural area of residence and income quintile to adjust for differences in baseline risk. The c-statistic was used to measure predictive validity of different models.

Third, we performed propensity score (PS) analyses in the Restricted Cohort. Since selection bias influences whether a person has a colonoscopy, comparing outcome events between subjects who had a colonoscopy and those who did not by means of conventional statistical modeling such as multivariate logistic analysis will still yield biased estimates of effect [16]. To adjust for the potentially confounding factors and selection bias due to non-random allocation of subjects to two groups, propensity scores [28,29] were calculated. The conditional probability that each person would have received a colonoscopy was estimated using multivariable logistic regression model, using the following as predictor variables: age, sex, neighborhood income quintile, co-morbidity index and rural residence. The c-statistic for the propensity score derivation model was 0.80, indicating good model discrimination. This model estimated the adjusted predicted probabilities (propensity scores) of colonoscopy use for each subject.

We explored three methods of propensity score analysis: (1) *Quintile stratification analysis* (Crude analysis of CRC outcomes based on PS quintile approach [18,30], stratifying subjects into 5 equal categories based on the propensity scores); (2) *Propensity score weighted analysis* (Using the “inverse probability of treatment weight [IPTW]” method [31,32], the PS was weighted by the inverse of the PS for subjects who had a colonoscopy and by the inverse of 1-PS for subjects without a colonoscopy. The probability of CRC incidence and mortality associated with colonoscopy use was estimated using a propensity score-weighted logistic regression model, controlling for other covariates.); and (3) *Propensity score matched analysis* (We used a greedy matching algorithm [33,34] to individually match persons receiving colonoscopy to unique persons who did not according to their propensity scores [35], yielding 86,592 matched pairs. Differences in the explanatory variables between those received and did not receive colonoscopy were tested using chi-square statistic. Standardized differences of the 2 groups were used to describe the treatment effect, with standardized differences greater than 10% considered meaningful [36]. CRC incidence and mortality differences between the two groups were estimated overall and for demographically defined subgroups using Kaplan-Meier survival curves. They were tested for their homogeneity with the Wilcoxon signed rank test for matched pairs. Differences in 7-year incidence and 5-year mortality rates were assessed using the McNemar test.)

Finally, we performed a fourth set of analyses using instrumental variables. Originally described in econometrics [19], instrumental variable analysis (IVA) is increasingly used in epidemiologic and health-service research studies [37-39] to balance the treatment groups to address hidden treatment selection bias. In theory, IVA addresses both the measured and unmeasured differences between the two groups. While the three previous analytic approaches considered measured confounders that might have influenced colonoscopy use, they could not account for any unmeasured factors. IVA estimates treatment effects in the presence of unmeasured hidden confounding [40,41], and is suited to health policy questions [42,43].

An instrumental variable should be strongly associated with the exposure, but not an independent determinant of the outcome of interest [44]. We used the PCP rate of discretionary colonoscopy as the instrumental variable for our analyses. “Discretionary colonoscopy” was defined as a colonoscopy procedure on a person without known risk factors for colorectal cancer, not performed during an inpatient stay, and not associated with a diagnosis of colorectal cancer at the time of colonoscopy or within a 3 year period following the colonoscopy. We assigned each PCP a rate of discretionary colonoscopy, defined as the number of discretionary colonoscopies per 100 eligible persons linked to him or her during 1996–2000. The PCP rate of discretionary colonoscopy has very useful properties as an instrument. It is highly correlated with receipt of colonoscopy and by definition is uncorrelated with CRC incidence and mortality, except for its association with colonoscopy. In the first stage of IV method, we predicted exposure to colonoscopy using the instrument and the subject level covariates. The predicted probability of receiving colonoscopy was then used as an independent variable in the second stage to predict CRC incidence and mortality, along with other subject level measured covariates. To account for the clustering of subject-level observations, parameters and standard errors were estimated robustly using a generalized estimating equation approach. The strength of the instrumental variable was examined using the OR and log-likelihood tests from first stage model, yielding a partial F-statistic of 10.

We reported the predicted probabilities in percentages with 95% confidence intervals for each statistical method used and absolute risk reduction ARR with 95% confidence intervals. P values less than 0.05 were considered statistically significant. All analyses were performed using SAS version 9.2.

## Results

### Comparison of outcomes in different cohorts

There were 1,341,612 subjects in the unselected cohort between the age of 50–74 years and 97,740 (7.3%) were exposed to a colonoscopy. There were 24,559 new cases and 5,556 deaths due to CRC from this cohort during the follow up period. Of the 1,089,998 subjects in the restricted cohort, 86,837 (8.0%) had a colonoscopy during the period 1996–2000. By definition, none of these colonoscopies resulted in a diagnosis of CRC. There were 14,455 (1.3%) new diagnosis of CRC and 2,394 (0.3%) deaths of CRC in the restricted cohort during the period.

Table 1 shows the observed probability for CRC incidence and mortality according to the receipt of colonoscopy in the two study cohorts. In the unselected cohort, the proportion of new cases of CRC in the colonoscopy group was 4.61% as compared with 1.61% in the no colonoscopy group, suggesting that colonoscopy increases the risk of CRC. Similarly, the proportion of death shows an increased likelihood of mortality among the colonoscopy users compared to non-users (1.16% vs. 0.36%). Clearly, there is no plausible explanation for why colonoscopy should be a risk factor for CRC. However, persons newly diagnosed with CRC will very commonly be exposed to colonoscopy, for the purpose of diagnosis, confirmation, or pre-operative planning. In this unselected cohort, subjects with colonoscopies that are performed for the diagnosis and symptoms of CRC were included, which could have resulted in this bias.

**Table 1:** Probability of CRC incidence and mortality in the two cohorts - Unadjusted model

**Study cohorts**		**Colonoscopy**	**Estimate**	**95% CI**	**Absolute risk difference**	**95% CI**
**Unselected cohort (n = 1,341,612)**						
	**Incidence**					
		No	1.61	1.59, 1.63	3.00	2.69, 3.30
		Yes	4.61	4.31,4.91		
	**Mortality**					
		No	0.36	0.33, 0.38	0.80	0.73, 0.87
		Yes	1.16	1.10, 1.23		
**Restricted cohort (n = 1,089,998)**						
	**Incidence**					
		No	1.36	1.35, 1.39	-0.52	-0.60, -0.46
		Yes	0.84	0.77, 0.91		
	**Mortality**					
		No	0.23	0.19, 0.27	-0.08	**-**0.13, -0.03
		Yes	0.15	0.12, 0.17		

Data are presented as percentages.

*CRC*, colorectal cancer; *CI*, confidence interval.

We then explored the possibility of this bias by analyzing the restricted cohort. The restricted cohort shows a reduction in incidence (0.84% vs. 1.36%) and mortality (0.15% vs. 0.23%) among the colonoscopy users compared to nonusers. There was an ARR of 0.52% for incidence and 0.08% for mortality in favor of colonoscopy. These results demonstrate how different methodologies in creating study cohorts change the causal relationship of treatment effect.

### Conventional regression analyses

Table 2 shows the predicted probabilities of CRC incidence and mortality for the two study cohorts using multivariate logistic regression with a GEE approach, adjusted for patient age, sex, neighborhood income quintile, comorbidity and rural residence. The predicted probabilities were calculated for each subject and then averaged for the population. The unselected cohort had an increased CRC incidence and mortality in the colonoscopy group even after adjusting for measured subject-level confounding factors, whereas the restricted cohort shows a reduction in CRC outcomes in the colonoscopy group. The remaining analyses were done only for the restricted cohort.

**Table 2:** Predicted probabilities of CRC incidence and mortality in the two cohorts - Multivariate logistic regression model

**Study cohorts**		**Colonoscopy**	**Estimate**	**95% CI**	**Absolute risk difference**	**95% CI**
**Unselected cohort (n = 1,341,612)**						
	**Incidence**					
		No	1.75	1.73, 1.77	3.26	3.22, 3.30
		Yes	5.01	4.97, 5.04		
	**Mortality**					
		No	0.38	0.33, 0.42	0.79	0.71, 0.87
		Yes	1.17	1.10, 1.23		
**Restricted cohort (n = 1,089,998)**						
	**Incidence**					
		No	1.42	1.39, 1.44	-0.53	-0.60, -0.44
		Yes	0.90	0.82, 0.98		
	**Mortality**					
		No	0.24	0.18, 0.29	-0.08	-0.19, 0.04
		Yes	0.16	0.13, 0.34		

Data are presented as percentages.

*CRC*, colorectal cancer; *CI*, confidence interval.

Multivariate logistic model adjusted subject characteristics such as age, sex, charlson comorbidity index, rural area of residence and income quintile.

### Propensity score analyses

#### Quintile stratification analysis

**Figure 2:**
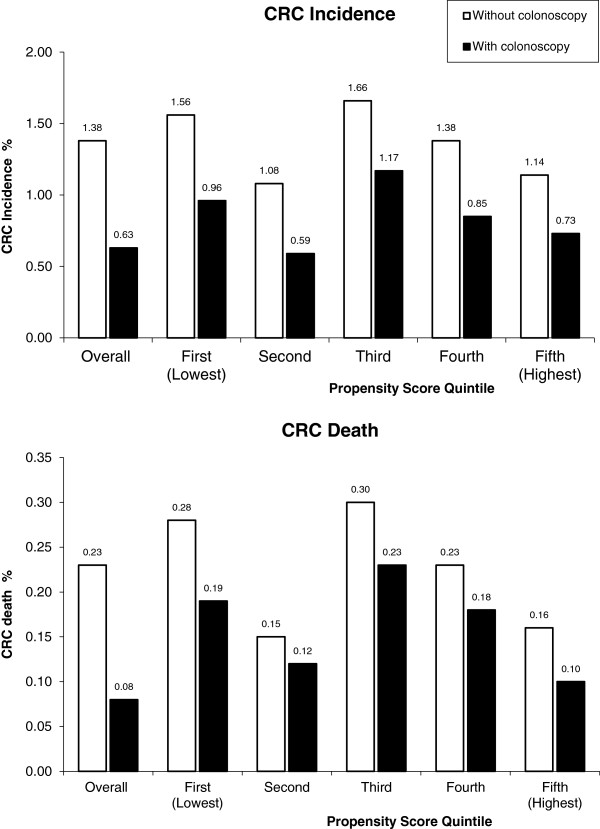
Comparison of incidence and mortality rate by subject colonoscopy status and propensity score quintiles – Restricted cohort.

Figure 2 shows the incidence and mortality rates by propensity score quintiles for colonoscopy use. In all the quintiles, the incidence rate and mortality rates were lower in patients who received colonoscopy than in those did not.

#### Propensity score matched analysis

Table 3 compares the baseline characteristics of subjects with and without colonoscopy in the restricted cohort and the PS matched cohort. In the restricted cohort, the colonoscopy users were more likely to be women, live in rural areas and areas with high neighborhood household income. The proportion of patients with comorbidity was higher in the colonoscopy group. The PS matched cohort with 86,592 colonoscopy users and nonusers, were similar with respect to all the measured characteristics.

**Table 3:** Comparison of baseline characteristics according to receipt of colonoscopy – Restricted cohort vs. PS Matched cohort

	**All patients**	**Restricted cohort**				**Propensity Matched sample**			
		**No colonoscopy**	**Yes colonoscopy**		**Standardized difference**	**No colonoscopy**	**Yes colonoscopy**		**Standardized difference**
**Characteristics**		**(%)**	**(%)**	**P value**		**(%)**	**(%)**	**P value**	
# of patients	1,089,998	1,003,161	86,837			86,592	86,592		
Female patients	591,431 (54.3)	543,040 (54.1)	48,391 (55.7)	<.001	3.22%	48,207 (55.7)	48,252 (55.7)	0.82	0.00%
Patient age group				<.001				0.62	
50-59	461,161 (42.3)	425,29 1 (42.4)	35,870 (41.3)		2.23%	35,695 (41.2)	35,775 (41.3)		0.20%
60-69	379,961 (34.9)	344,492 (34.3)	35,469 (40.9)		13.66%	35,289 (40.8)	35,365 (40.8)		0.00%
70-74	248,876 (22.8)	233,378 (23.3)	15,498 (17.9)		13.38%	15,608 (18.0)	15,452 (17.8)		0.52%
Income quintile				<.001				0.75	
01	187,006 (17.2)	173,598 (17.3)	13,408 (15.1)		5.97%	13,230 (15.3)	13,407 (15.5)		0.55%
02	219,705 (20.1)	203,403 (20.3)	16,302 (18.7)		4.04%	16,378 (18.9)	16,300 (18.8)		0.26%
03	220,765 (20.3)	203,512 (20.3)	17,253 (19.9)		1.00%	17,326 (20.0)	17,251 (19.9)		0.25%
04	219,289 (20.1)	201,626 (20.1)	17,663 (20.5)		0.99%	17,587 (20.3)	17,663 (20.4)		0.25%
05	240,663 (22.1)	218,691 (21.8)	21,972 (25.7)		9.17%	22,071 (25.5)	21,971 (25.4)		0.23%
Missing	2,570 (0.3)	2,331 (0.2)	239 (0.3)						
Rural area of residence	149,199 (13.7)	136,594 (13.6)	12,605 (14.5)	<.001	2.59%	11,262 (13.0)	12,434 (14.4)	<.001	4.07%
Co-morbidities				<.001				0.13	
Not hospitalized Hospitalized but no comorbidities	519,817 (47.7)	514,294 (51.3)	5,523 (6.4)		114.09%	5,516 (6.4)	5,516 (6.4)		0.00%
	401,168 (36.8)	337,586 (33.6)	63,582 (73.2)		86.49%	63,752 (73.6)	63,415 (73.2)		0.91%
At least one comorbidity	169,013 (15.5)	151,281 (15.1)	17,732 (20.4)		13.90%	17,324 (20.0)	17,661 (20.4)		1.00%

Data are presented as number (percentage). Standardized difference is the mean difference divided by the pooled SD, expressed as a percentage.

**Figure 3:**
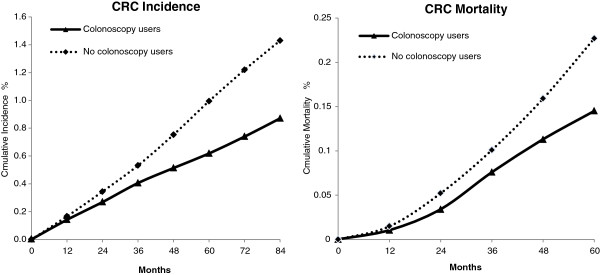
**Estimates of cumulative Incidence and Mortality Rates for Propensity Matched Cohort.** Note: Differences in cumulative rates between colonoscopy user and nonusers at each time point shown after 12 months were significant (p < 0.001).

Figure 3 shows the cumulative CRC incidence and mortality for the PS matched cohort. The colonoscopy users were less likely to have developed or died of CRC at each time point. For example, cumulative incidence rates were 0.14% in colonoscopy users versus 0.17% in nonusers at 1 year and 0.87% versus 1.43% at 5 years. The mortality relative reduction was 15% at 1 year and 39% at 5 years (P < 0.001).

Table 4 shows the cumulative CRC incidence and mortality among colonoscopy users and matched nonusers for the PS matched cohort according to age and sex. All groups had lower incidence and mortality among colonoscopy users than among matched nonusers. The incidence and mortality reductions increased with age and were greater in men than women in each age group. The cumulative reduction in 7-year incidence and 5-year mortality was greater in men than women. There was a greater reduction in incidence and mortality in lower income quintiles.

**Table 4:** Cumulative CRC incidence and mortality rates for PS matched cohort

	**Cumulative incidence rates**			**Cumulative mortality rates**		
**Participant groups**	**Colonoscopy users**	**Nonusers**	**Difference**	**Colonoscopy users**	**Nonusers**	**Difference**
**All matched pairs**	**0.87%**	**1.43%**	**0.56%**	**0.15%**	**0.23%**	**0.08%**
**By Sex and age group**						
**Men**	**0.99%**	**1.81%**	**0.82%**	**0.16%**	**0.29%**	**0.13%**
Age 50-S9y	0.55%	1.10%	0.56%	0.11%	0.14%	0.03%
Age 60-69y	1.16%	1.93%	0.77%	0.17%	0.32%	0.15%
Age 70-74y	1.72%	3.41%	1.69%	0.27%	0.62%	0.35%
**Women**	**0.78%**	**1.13%**	**0.35%**	**0.14%**	**0.18%**	**0.05%**
Age 50-59y	0.40%	0.54%	0.14%	0.05%	0.04%	0.01%
Age 60-69y	0.97%	1.36%	0.39%	0.19%	0.22%	0.03%
Age 70-74y	1.26%	2.07%	0.81%	0.22%	0.46%	0.24%
**By lncome quintile**						
Q1	0.91%	1.59%	0.68%	0.14%	0.32%	0.18%
Q2	0.83%	1.55%	0.72%	0.17%	0.28%	0.11%
Q3	0.97%	1.41%	0.44%	0.18%	0.22%	0.05%
Q4	0.86%	1.37%	0.51%	0.12%	0.20%	0.07%
Q5	0.81%	1.31%	0.50%	0.13%	0.18%	0.06%

*CRC*, colorectal cancer; *PS*, propensity score.

#### Propensity score weighted analysis

The predicted probabilities for both incidence (Table 5) and mortality (Table 6) using PS weighted outcome analysis were higher than the unadjusted model suggesting the effect of confounders in the model.. However, the absolute risk differences were similar to the unadjusted model.

**Table 5:** Risk reductions in CRC incidence § associated with colonoscopy - comparison of different methods

**Model**	**Predicted probability (95% Cl)**		**Absolute risk reduction (95% Cl)**
	**No colonoscopy**	**Any colonoscopy**	
Unadjusted Model	1.36 (1.35, 139)	0.84 (0.77, 0.91)	-0.52 (-0.60, -0.46)
Multivariate Logistic Regression Model†	1.42 (1.39, 1.44)	0.90 (0.82, 0.98)	-0.53 (-0.60, -0.44)
Propensity Score Weighted Model‡	1.40 (1.37, 1.42)	0.86 (0.83, 0.89)	-0.54 (-0.69, -0.39)
Propensity Score Matched Model∞	1.43 (1.33, 1.52)	0.87 (0.76, 0.98)	-0.56 (-0.99, -0.12)
IV Adjusted Model**∂**	1.25 (1.19, 1.30)	0.65 (0.41, 0.99)	-0.60 (-0.78, -0.31)

Data are presented as percentages.

*CRC*, colorectal cancer; *CI*, confidence interval.

† Multivariate logistic model adjusted subject characteristics such as age, sex, charlson comorbidity index, rural area of residence and income quintile.

‡ Propensity score weight calculated as 1/ps for colonoscopy users and 1/(1-ps) for the non users.

∞ PS matched model on 86,592 pairs.

∂ IV adjusted probabilities were estimated using 2-stage probit models. In the first stage, the probability of receiving colonoscopy was modeled as a function of the PCP rate of colonoscopy. In the second stage, the probability of CRC incidence and mortality was modeled using the predicted probability of colonoscopy as an independent variable, adjusted for PCP and subject characteristics.

§ Incidence up to 7 years.

**Negative values indicate a reduction in the absolute risk of CRC incidence.

**Table 6:** Risk reductions in CRC mortality § associated with colonoscopy -comparison of different methods

**Model**	**Predicted probability (95% Cl)**		**Absolute risk reduction (95% Cl)**
	**No colonoscopy**	**Any colonoscopy**	
Unadjusted Model	0.23 (0.19, 0.27)	0.15 (0.12, 0.17)	-0.08 (-0.13, -0.03)
Multivariate Logistic Regression Model†	0.24 (0.18, 0.29)	0.16 (0.13, 0.34)	-0.08 (-0.19, -0.04)
Propensity Score Weighted Model‡	0.25 (0.20, 0.29)	0.17 (0.11, 0.23)	-0.08 (-0.15, -0.02)
Propensity Score Matched Model∞	0.27 (0.05, 0.49)	0.17 (0.01, 0.41)	-0.10 (-1.24, -1.04)
IV Adjusted Model**∂**	0.21 (0.17, 0.26)	0.04 (0.01, 0.12)	-0.17 (-0.21, -0.14)

Data are presented as percentages.

*CRC*, colorectal cancer; *CI*, confidence interval.

† Multivariate logistic model adjusted subject characteristics such as age, sex, charlson comorbidity index, rural area of residence and income quintile.

‡ Propensity score weight calculated as 1/ps for colonoscopy users and 1/(1-ps) for the non users.

∞ PS matched model on 86,592 pairs.

∂ IV adjusted probabilities were estimated using 2-stage probit models. In the first stage, the probability of receiving colonoscopy was modeled as a function of the PCP rate of colonoscopy. In the second stage, the probability of CRC incidence and mortality was modeled using the predicted probability of colonoscopy as an independent variable, adjusted for subject characteristics.

§ Mortality up to 5 years.

**Negative values indicate a reduction in the absolute risk of CRC mortality.

### Instrumental variable analyses

Using IVA, the predicted probability for CRC incidence in the colonoscopy and no colonoscopy groups were 0.65% and 1.25% respectively, which were lower than the unadjusted estimates (Table 5). Similarly the predicted probabilities for mortality were lower in the IVA model (Table 6). The corresponding ARR was 0.60% (95% CI: -0.78, -0.31) for incidence and 0.17% (95% CI: -0.21, -0.14) for mortality, higher than the unadjusted model.

### Comparison of methods

Table 5 also compares the reduction in CRC incidence associated with colonoscopy for the restricted cohort using different statistical methods. The predicted probabilities for CRC incidence in the logistic model, PS weighted outcome model and PS matched models were higher than the unadjusted model, whereas the IVA estimates were lower than the unadjusted model. (Table 5) The absolute risk reduction in an unadjusted analysis was 0.52% (95% CI; 0.60%, 0.46%) for CRC incidence. With adjustment for age, sex, income quintile, rural area of residence and comorbidity using logistic regression the ARR increased slightly to 0.53%, but did not change meaningfully. Next, the propensity score weighted outcome model and PS matched analysis produced slightly higher risk reductions with ARR of 0.54% and 0.56% respectively. The final model using IVA method produced an ARR of 0.60% suggesting the effect of hidden confounding in the model.

Table 6 compares the reduction in CRC mortality associated with colonoscopy for the restricted cohort using different statistical methods. The predicted probabilities for CRC mortality were higher in all models except IVA compared to the unadjusted model. The ARR for CRC mortality was 0.08% for unadjusted model, multivariate model and for PS weighted outcome model. It increased up to 0.17% using the IVA model with a 95% CI of -0.21, -0.14. The reductions for both incidence and mortality were larger in IVA than other analyses. This was expected as the predicted probability of exposure changed from a dichotomous level to a range of values from 0 to 1. Also, IVA yields an ARR that applies to the marginal population, which is expected to be different than the average population.

## Discussion

We studied methodological approaches to addressing selection and measurement bias in a population-based study of the effectiveness of colonoscopy use in the prevention of CRC incidence and mortality. First, we compared the outcomes in an unselected cohort of subjects in whom exposure to colonoscopy was measured during the same time period that outcomes were ascertained, and also among subjects in a restricted cohort when exposure was not measured during the same period of time that outcome events were measured. The unselected cohort showed a paradoxical increase in CRC incidence (4.61% vs. 1.61%) and mortality (1.16% vs. 0.36%) associated with colonoscopy use, whereas the restricted cohort showed a reduction in incidence (0.84% vs. 1.37%) and mortality (0.15% vs. 0.23%) among colonoscopy users compared to nonusers.

We then explored different methods of analysis of the restricted cohort, using a conventional regression model, propensity score analysis, and instrumental variable analysis, to adjust for potential confounding factors and selection bias due to non-random allocation of study subjects. For CRC incidence, the absolute risk reduction associated with colonoscopy use was 0.52% in an unadjusted model, 0.53% in a multivariate logistic model, 0.54% in a propensity score-weighted outcome model, 0.56% in propensity score-matched model, and 0.60% using instrumental variable analysis. For CRC mortality, the ARR was 0.08% in the unadjusted model, multivariate model and for a propensity score- weighted outcome model, 0.10% in propensity score-matched model and 0.17% using the IVA model. Using all the above models, we found that colonoscopy was beneficial in reducing the risk of CRC incidence and mortality in the restricted cohort even after adjustment for important biases such as treatment selection and hidden biases.

The different results between the two study cohorts are likely due to measurement error. When we looked at the subjects in unselected cohort that were not in the restricted cohort, we found that these were subjects with a colonoscopy with a subsequent diagnosis of CRC and might be with some clinical indication. This underscores the challenge of not being able to differentiate screening from diagnostic tests. In the unselected group, colonoscopies performed for the diagnosis of symptoms and signs of colorectal cancer could not be excluded and inclusion of these patients resulted in bias. The increased risk of CRC among patients who had colonoscopy in the unselected cohort is mostly attributable to inclusion of colonoscopies performed in persons with symptoms of CRC.

In subsequent analyses using only the restricted cohort, we found reductions in 7-year incidence and 5-year mortality associated with colonoscopy using all models, even though the ARR did not vary much for all methods other than the IVA.

The standard multivariate logistic regression method assumes that the relationship between the risk factors and outcome are correctly specified in the model and all relevant baseline risk factors have been measured. However, in many situations these assumptions are not met because it is impossible to measure all important risk factors. It is also difficult to know whether the relationships between risk factors and outcome are correctly specified in the model. The results from this standard analysis can be used to compare the results from other method for model misspecification and unmeasured confounding.

Propensity score quintile analysis, which is another way to address potential model misspecification and selection bias, is less parametric compared to standard regression. This method compares the outcomes across different groups of patients with similar propensity to receive the colonoscopy. Stratification based on quintiles of propensity scores removes more than 90% of the bias [29]. This method attempts to balance the observed characteristics in the exposed groups as would occur in a randomized experiment [45]. The method is drawn from the same discipline of standard regression which assumes that all relevant baseline risk factors have been measured and correctly specified in the model. However, in many situations these assumptions are not met and there can be unmeasured confounders that can affect the exposure of interest.

The Propensity score weighted analysis produced similar estimates of the standard method suggesting that the standard model has adjusted correctly for any differences in measured covariates. The Propensity score weighted analysis uses the inverse of the propensity score to weight each observation in the exposed group, and one minus the inverse of the propensity score (i.e., the propensity of NOT being in the treated group) in the non exposed group. Weighting has the advantage of including all the data (unless weights are set to 0) and does not depend on sampling or matching. While this method has useful mathematical properties, it does not work well in practice. If an exposed subject happens to have a very low probability of being treated, the value of the inverse of the propensity score will be extremely high, asymptotically infinity [31]. The effect size obtained will be dominated by this single value, and any fluctuations in it will produce wildly varied results, which is an undesirable property.

The PS matching method produced an absolute reduction of 0.56% for CRC incidence and 0.10% for CRC mortality, which are larger than the standard method. However, the confidence intervals are very wide and overlap the confidence regions. The reduced sample size due to matching could lead to less précised results. Matching is a method for sampling a large reservoir of potential controls to produce a control group of modest size that is ostensibly similar to the treated group. In practice, there is a trade-off between the desires to find matches for all treated units and to obtain matched treated-control pairs that are extremely similar to each other. While the PS matching method addresses the overt bias, it does not address or resolve confounding due to unmeasured factors. It also leaves a small residual bias due to inexact matching.

Our IVA findings highlighted the important role of PCP in cancer screening. The inadequacy of adjustment for only measured confounders is evidenced by the fact that the IVA estimate of the absolute difference of 0.60% for CRC incidence was larger than other methods. Similarly, the ARR for CRC mortality was 0.08% for the standard model, and it increased up to 0.17% using the IVA model. The confidence intervals are wider than the standard model which is expected as the IV approach depends on much less variation in user status [16]. We suspect that there is bias coming from the unmeasured variables that are associated with physicians’ use of colonoscopy. In our previous study we showed there are significant variations with respect to PCP use of colonoscopy [14]. Another reason for the difference in estimates is that IVA calculates only the marginal effect on the population under study, which differs from the average effect. The marginal population [43] excludes persons who would “always” or “never” receive colonoscopy, focusing on patients whose indication for colonoscopy is more discretionary. Hence the reduction is for the intermediate risk group for whom colonoscopy is not required for complications or symptoms due to CRC yet the benefit of colonoscopy is uncertain. Thus, IVA is well suited for policy questions, rather than addressing the effect on a particular patient. Its external validity depends on the population studied. The variable we used-PCP rate of discretionary colonoscopy- meets the assumptions of a valid instrument as PCP’s practice of screening colonoscopy is highly correlated with the individual level receipt of colonoscopy among their subjects. In addition, because of the way we defined discretionary colonoscopy—by definition, a discretionary colonoscopy cannot occur in a patient who subsequently develops CRC—a PCP’s rate of discretionary colonoscopy is not independently associated with the risk of CRC among his or her patients.

The challenge in IVA is to identify a good valid instrument. It is often conceptually credible to assume the lack of association between the instrument and outcome, but impossible to verify empirically or statistically. We explored other instruments for use in the study, such as geographical region of residence, time period, and intensity of health services use. The PCP rate of discretionary colonoscopy was the variable with the best theoretical attributes of an instrumental variable, and was associated with the most variation in the receipt of colonoscopy.

Our study has several limitations. First, we have not included the 25% of the Ontario population without a usual provider of care. These subjects may have a very low rate of colonoscopy as well as CRC screening, as they do not have an opportunity to receive such recommendations from their PCP [46]. Second, because of the cross-classified structure of the data, patients may not be completely nested within physicians. To address this limitation, we included only patients who had the same PCP over the 5 year period from 1996–2000. Third, our definition of COC required patients to have at least 2 visits with a PCP per year to be linked to a PCP. We did not include the small number of apparently healthy patients who had only 1 or no visits per year. Fourth, some potential confounders were not directly measured in our study, such as a family history of CRC. Fifth, we did not consider other types of screening in our study, such as fecal occult blood test or flexible sigmoidoscopy. Because we focused only in the potential benefits of colonoscopy and not the potential harms, any estimates of the overall benefit of colonoscopy should explicitly incorporate both benefits and risks. Finally, we did not account for variation in technique of colonoscopy. The impact of colonoscopy in the diagnosis of CRC depends on the quality of the examination. In our study, 83% of all colonoscopies were classified as complete. Overall, however, the limitations of our study design do not substantially affect our ability to accomplish our principal research objective of estimating the population level effect of colonoscopy. Our results are estimates of the effect of colonoscopy use in typical practice settings. Our findings could help other researchers in the design and analysis of studies which test other screening methods.

## Conclusion

In conclusion different methods of subject selection and statistical analysis provided different estimates of the effectiveness of colonoscopy. It is very important to understand the sources of bias and how design and analytic strategies can impact study findings, particularly with the use of administrative data for evaluating screening modalities.

## Competing interests

The authors declare that they have no competing interests.

## Authors’ contributions

BJJ was responsible for the data collection, analyses and interpretation of data, drafting the manuscript and the process of seeking funding. NNB made significant contributions to interpretations of data and also took part in editing the manuscript. RM and RS were the resource persons for statistical analysis, assisted with interpretation of the data and helped in editing the manuscript. DRU was responsible for the conception and design of study, and manuscript finalizing. All of the authors read and approved the final manuscript.

## Pre-publication history

The pre-publication history for this paper can be accessed here:

http://www.biomedcentral.com/1471-2288/13/59/prepub
